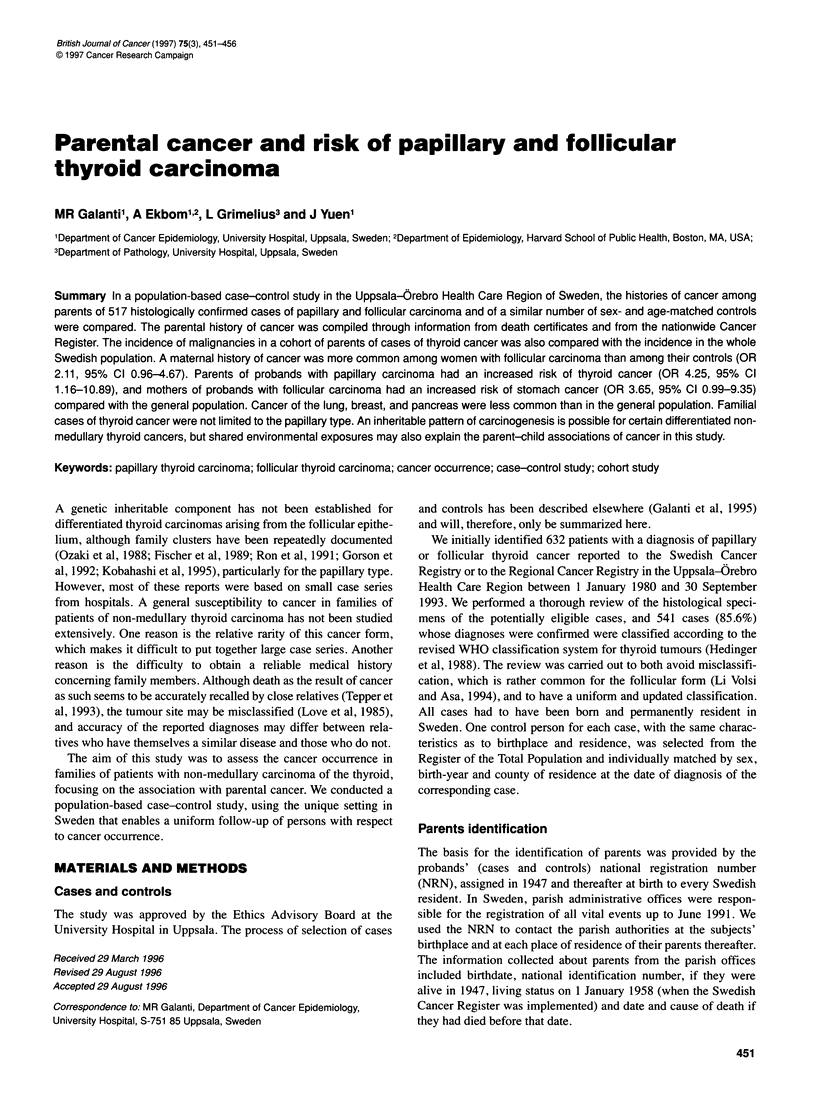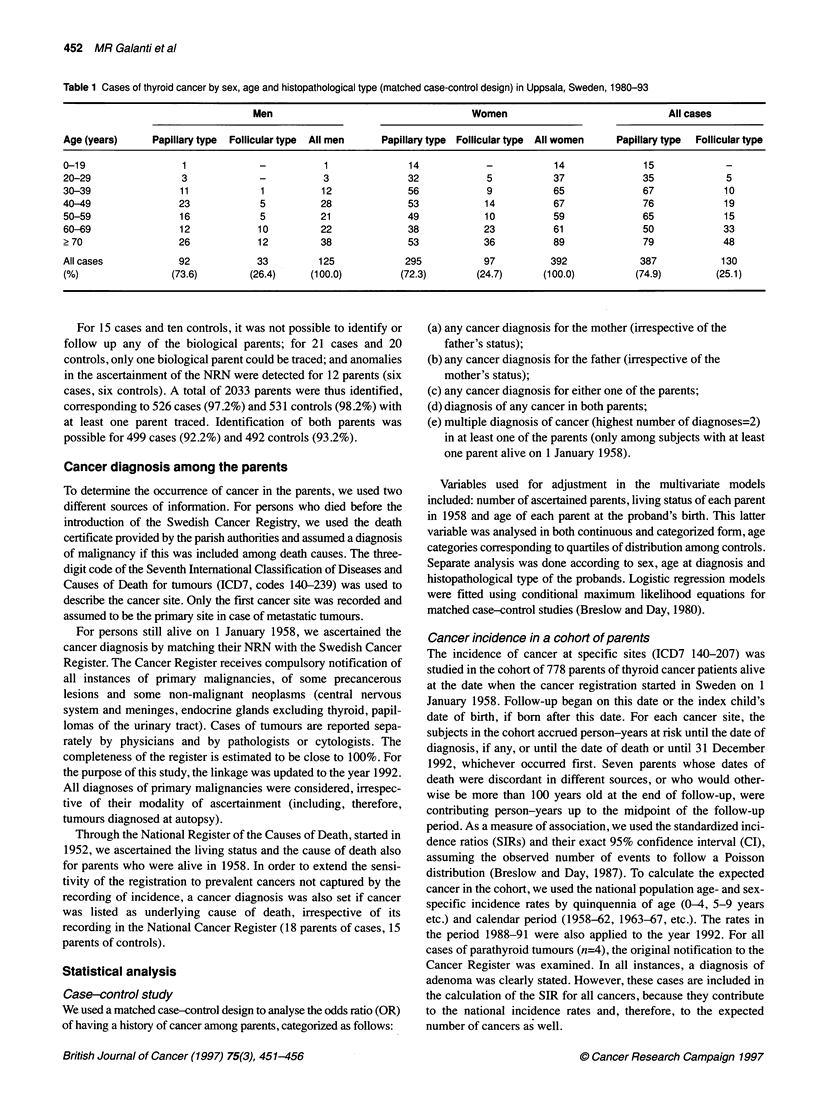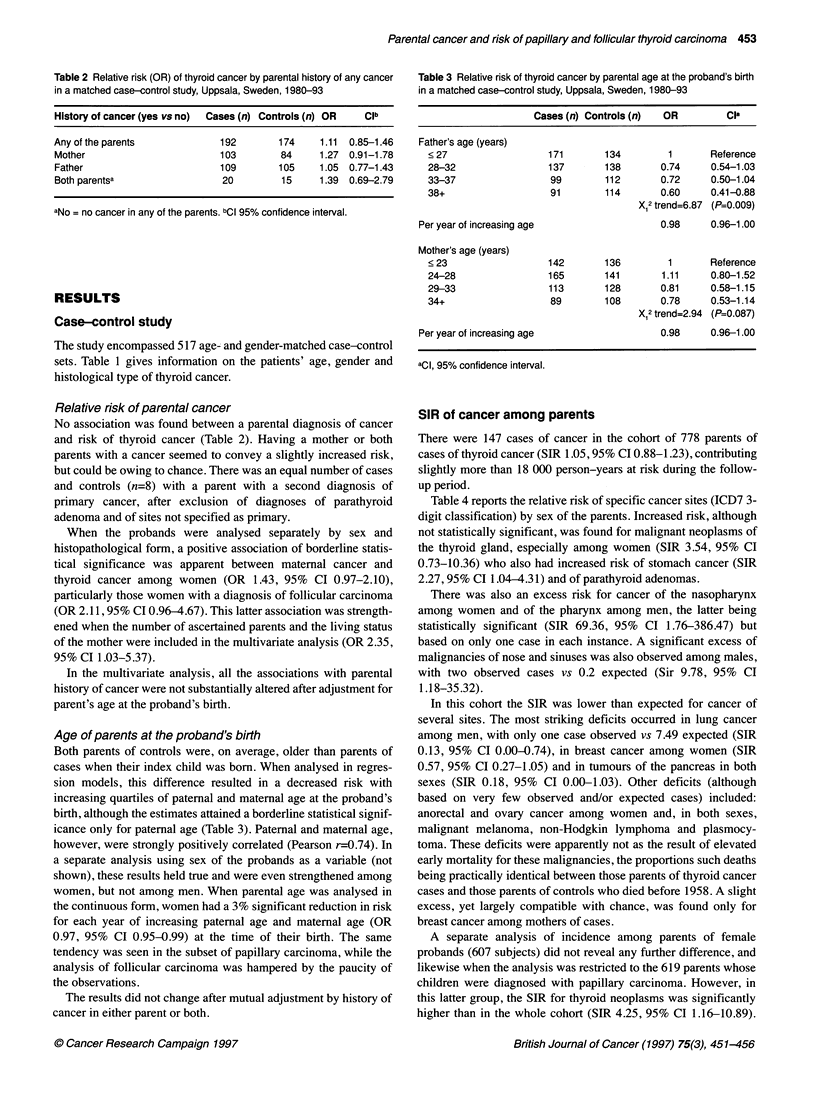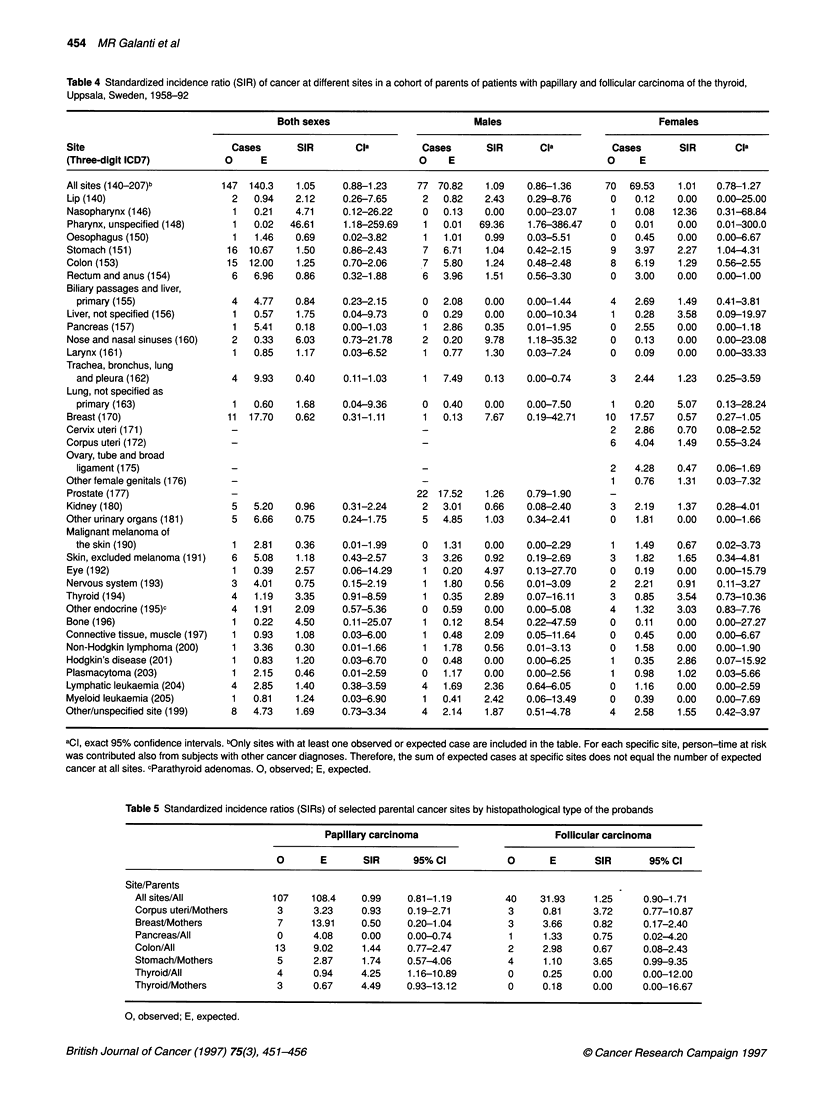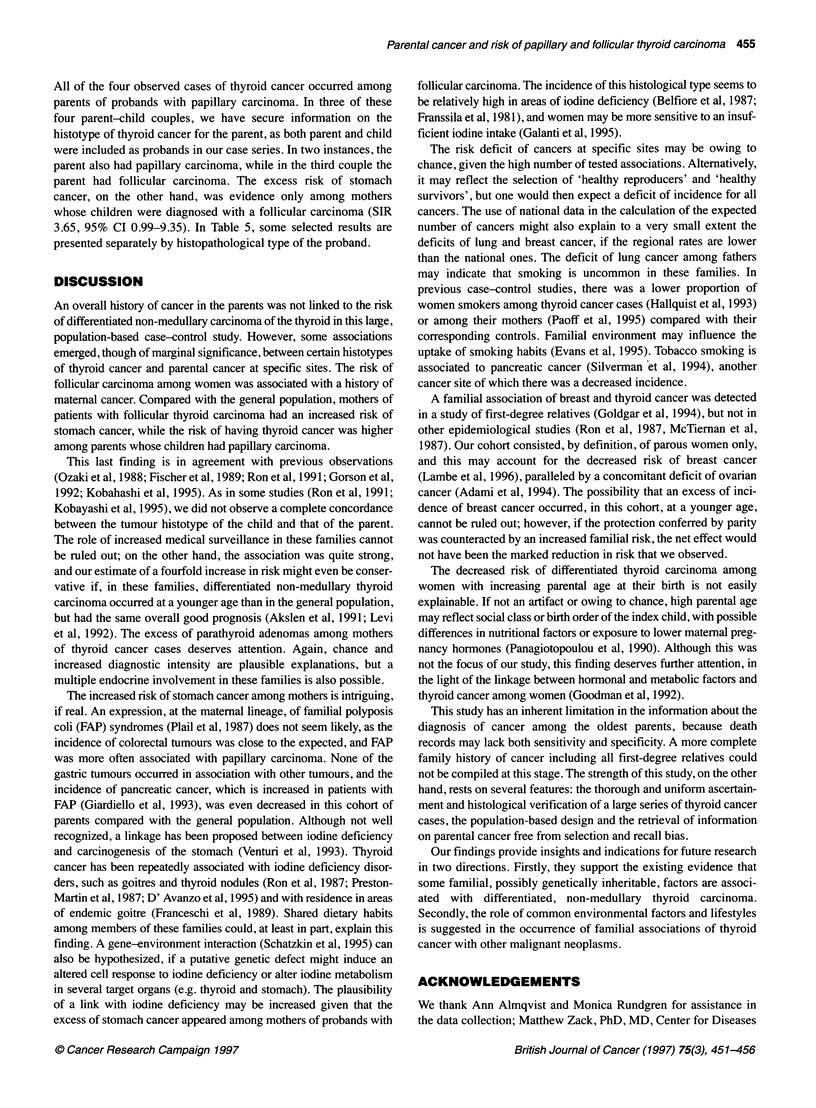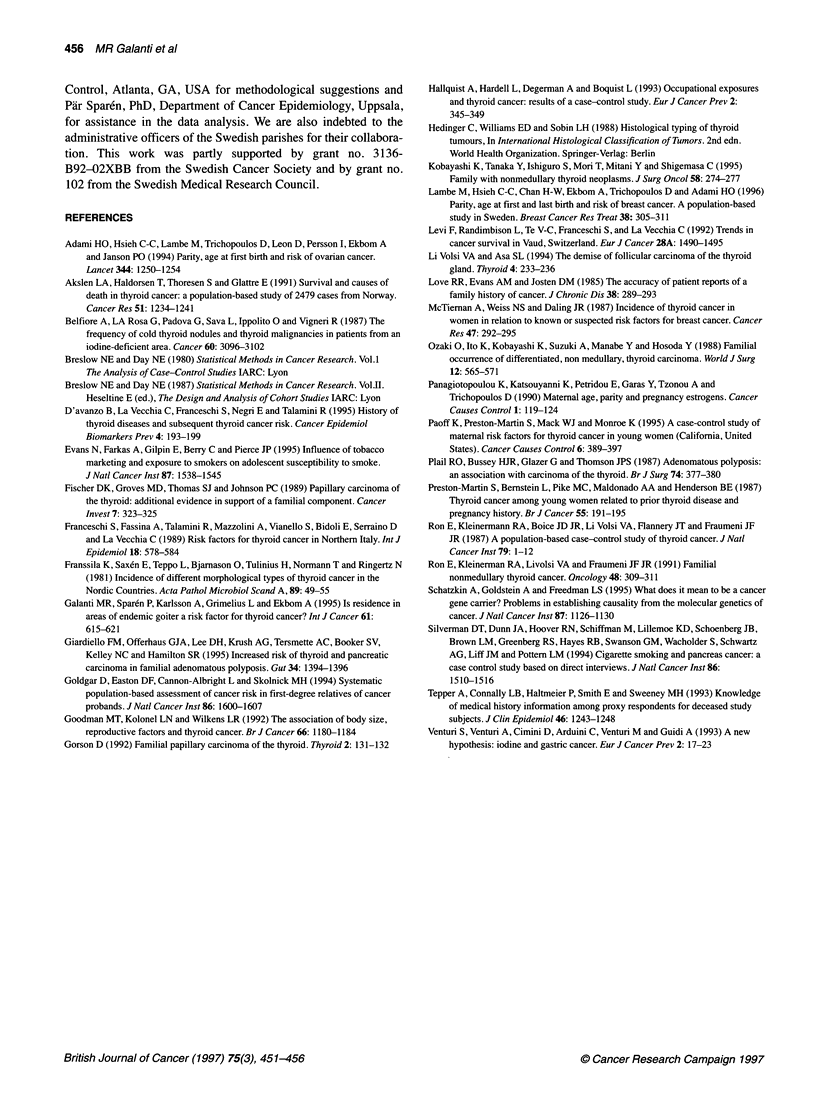# Parental cancer and risk of papillary and follicular thyroid carcinoma.

**DOI:** 10.1038/bjc.1997.76

**Published:** 1997

**Authors:** M. R. Galanti, A. Ekbom, L. Grimelius, J. Yuen

**Affiliations:** Department of Cancer Epidemiology, University Hospital, Uppsala, Sweden.

## Abstract

In a population-based case-control study in the Uppsala-Orebro Health Care Region of Sweden, the histories of cancer among parents of 517 histologically confirmed cases of papillary and follicular carcinoma and of a similar number of sex- and age-matched controls were compared. The parental history of cancer was compiled through information from death certificates and from the nationwide Cancer Register. The incidence of malignancies in a cohort of parents of cases of thyroid cancer was also compared with the incidence in the whole Swedish population. A maternal history of cancer was more common among women with follicular carcinoma than among their controls (OR 2.11, 95% CI 0.96-4.67). Parents of probands with papillary carcinoma had an increased risk of thyroid cancer (OR 4.25, 95% CI 1.16-10.89), and mothers of probands with follicular carcinoma had an increased risk of stomach cancer (OR 3.65, 95% CI 0.99-9.35) compared with the general population. Cancer of the lung, breast, and pancreas were less common than in the general population. Familial cases of thyroid cancer were not limited to the papillary type. An inheritable pattern of carcinogenesis is possible for certain differentiated non-medullary thyroid cancers, but shared environmental exposures may also explain the parent-child associations of cancer in this study.


British Journal of Cancer (1997) 75(3), 451–456
© 1997 Cancer Research Campaign

### Parental cancer and risk of papillary and follicular thyroid carcinoma

**MR Galanti[1], A Ekbom[1,2], L Grimelius[3] and J Yuen[1]**

[1]Department of Cancer Epidemiology, University Hospital, Uppsala, Sweden; [2]Department of Epidemiology, Harvard School of Public Health, Boston, MA, USA; [3]Department of Pathology, University Hospital, Uppsala, Sweden

**Summary** In a population-based case–control study in the Uppsala–Örebro Health Care Region of Sweden, the histories of cancer among parents of 517 histologically confirmed cases of papillary and follicular carcinoma and of a similar number of sex- and age-matched controls were compared. The parental history of cancer was compiled through information from death certificates and from the nationwide Cancer Register. The incidence of malignancies in a cohort of parents of cases of thyroid cancer was also compared with the incidence in the whole Swedish population. A maternal history of cancer was more common among women with follicular carcinoma than among their controls (OR 2.11, 95% CI 0.96–4.67). Parents of probands with papillary carcinoma had an increased risk of thyroid cancer (OR 4.25, 95% CI 1.16–10.89), and mothers of probands with follicular carcinoma had an increased risk of stomach cancer (OR 3.65, 95% CI 0.99–9.35) compared with the general population. Cancer of the lung, breast, and pancreas were less common than in the general population. Familial cases of thyroid cancer were not limited to the papillary type. An inheritable pattern of carcinogenesis is possible for certain differentiated non-medullary thyroid cancers, but shared environmental exposures may also explain the parent–child associations of cancer in this study.

**Keywords:** papillary thyroid carcinoma; follicular thyroid carcinoma; cancer occurrence; case–control study; cohort study

A genetic inheritable component has not been established for differentiated thyroid carcinomas arising from the follicular epithelium, although family clusters have been repeatedly documented (Ozaki et al, 1988; Fischer et al, 1989; Ron et al, 1991; Gorson et al, 1992; Kobahashi et al, 1995), particularly for the papillary type. However, most of these reports were based on small case series from hospitals. A general susceptibility to cancer in families of patients of non-medullary thyroid carcinoma has not been studied extensively. One reason is the relative rarity of this cancer form, which makes it difficult to put together large case series. Another reason is the difficulty to obtain a reliable medical history concerning family members. Although death as the result of cancer as such seems to be accurately recalled by close relatives (Tepper et al, 1993), the tumour site may be misclassified (Love et al, 1985), and accuracy of the reported diagnoses may differ between relatives who have themselves a similar disease and those who do not.

The aim of this study was to assess the cancer occurrence in families of patients with non-medullary carcinoma of the thyroid, focusing on the association with parental cancer. We conducted a population-based case–control study, using the unique setting in Sweden that enables a uniform follow-up of persons with respect to cancer occurrence.

### MATERIALS AND METHODS

### Cases and controls

The study was approved by the Ethics Advisory Board at the University Hospital in Uppsala. The process of selection of cases and controls has been described elsewhere (Galanti et al, 1995) and will, therefore, only be summarized here.

We initially identified 632 patients with a diagnosis of papillary or follicular thyroid cancer reported to the Swedish Cancer Registry or to the Regional Cancer Registry in the Uppsala–Örebro Health Care Region between 1 January 1980 and 30 September 1993. We performed a thorough review of the histological specimens of the potentially eligible cases, and 541 cases (85.6%) whose diagnoses were confirmed were classified according to the revised WHO classification system for thyroid tumours (Hedinger et al, 1988). The review was carried out to both avoid misclassification, which is rather common for the follicular form (Li Volsi and Asa, 1994), and to have a uniform and updated classification. All cases had to have been born and permanently resident in Sweden. One control person for each case, with the same characteristics as to birthplace and residence, was selected from the Register of the Total Population and individually matched by sex, birth-year and county of residence at the date of diagnosis of the corresponding case.

### Parents identification

The basis for the identification of parents was provided by the probands' (cases and controls) national registration number (NRN), assigned in 1947 and thereafter at birth to every Swedish resident. In Sweden, parish administrative offices were responsible for the registration of all vital events up to June 1991. We used the NRN to contact the parish authorities at the subjects' birthplace and at each place of residence of their parents thereafter. The information collected about parents from the parish offices included birthdate, national identification number, if they were alive in 1947, living status on 1 January 1958 (when the Swedish Cancer Register was implemented) and date and cause of death if they had died before that date.

*Received 29 March 1996*
*Revised 29 August 1996*
*Accepted 29 August 1996*

*Correspondence to:* MR Galanti, Department of Cancer Epidemiology, University Hospital, S-751 85 Uppsala, Sweden

**Table 1** Cases of thyroid cancer by sex, age and histopathological type (matched case-control design) in Uppsala, Sweden, 1980–93

| | Men | | | Women | | | All cases | |
|---|---|---|---|---|---|---|---|---|
| Age (years) | Papillary type | Follicular type | All men | Papillary type | Follicular type | All women | Papillary type | Follicular type |
| 0–19 | 1 | – | 1 | 14 | – | 14 | 15 | – |
| 20–29 | 3 | – | 3 | 32 | 5 | 37 | 35 | 5 |
| 30–39 | 11 | 1 | 12 | 56 | 9 | 65 | 67 | 10 |
| 40–49 | 23 | 5 | 28 | 53 | 14 | 67 | 76 | 19 |
| 50–59 | 16 | 5 | 21 | 49 | 10 | 59 | 65 | 15 |
| 60–69 | 12 | 10 | 22 | 38 | 23 | 61 | 50 | 33 |
| ≥ 70 | 26 | 12 | 38 | 53 | 36 | 89 | 79 | 48 |
| All cases | 92 | 33 | 125 | 295 | 97 | 392 | 387 | 130 |
| (%) | (73.6) | (26.4) | (100.0) | (72.3) | (24.7) | (100.0) | (74.9) | (25.1) |

For 15 cases and ten controls, it was not possible to identify or follow up any of the biological parents; for 21 cases and 20 controls, only one biological parent could be traced; and anomalies in the ascertainment of the NRN were detected for 12 parents (six cases, six controls). A total of 2033 parents were thus identified, corresponding to 526 cases (97.2%) and 531 controls (98.2%) with at least one parent traced. Identification of both parents was possible for 499 cases (92.2%) and 492 controls (93.2%).

### Cancer diagnosis among the parents

To determine the occurrence of cancer in the parents, we used two different sources of information. For persons who died before the introduction of the Swedish Cancer Registry, we used the death certificate provided by the parish authorities and assumed a diagnosis of malignancy if this was included among death causes. The three-digit code of the Seventh International Classification of Diseases and Causes of Death for tumours (ICD7, codes 140–239) was used to describe the cancer site. Only the first cancer site was recorded and assumed to be the primary site in case of metastatic tumours.

For persons still alive on 1 January 1958, we ascertained the cancer diagnosis by matching their NRN with the Swedish Cancer Register. The Cancer Register receives compulsory notification of all instances of primary malignancies, of some precancerous lesions and some non-malignant neoplasms (central nervous system and meninges, endocrine glands excluding thyroid, papillomas of the urinary tract). Cases of tumours are reported separately by physicians and by pathologists or cytologists. The completeness of the register is estimated to be close to 100%. For the purpose of this study, the linkage was updated to the year 1992. All diagnoses of primary malignancies were considered, irrespective of their modality of ascertainment (including, therefore, tumours diagnosed at autopsy).

Through the National Register of the Causes of Death, started in 1952, we ascertained the living status and the cause of death also for parents who were alive in 1958. In order to extend the sensitivity of the registration to prevalent cancers not captured by the recording of incidence, a cancer diagnosis was also set if cancer was listed as underlying cause of death, irrespective of its recording in the National Cancer Register (18 parents of cases, 15 parents of controls).

### Statistical analysis

### *Case–control study*

We used a matched case–control design to analyse the odds ratio (OR) of having a history of cancer among parents, categorized as follows:

(a) any cancer diagnosis for the mother (irrespective of the father's status);
(b) any cancer diagnosis for the father (irrespective of the mother's status);
(c) any cancer diagnosis for either one of the parents;
(d) diagnosis of any cancer in both parents;
(e) multiple diagnosis of cancer (highest number of diagnoses=2) in at least one of the parents (only among subjects with at least one parent alive on 1 January 1958).

Variables used for adjustment in the multivariate models included: number of ascertained parents, living status of each parent in 1958 and age of each parent at the proband's birth. This latter variable was analysed in both continuous and categorized form, age categories corresponding to quartiles of distribution among controls. Separate analysis was done according to sex, age at diagnosis and histopathological type of the probands. Logistic regression models were fitted using conditional maximum likelihood equations for matched case–control studies (Breslow and Day, 1980).

### *Cancer incidence in a cohort of parents*

The incidence of cancer at specific sites (ICD7 140–207) was studied in the cohort of 778 parents of thyroid cancer patients alive at the date when the cancer registration started in Sweden on 1 January 1958. Follow-up began on this date or the index child's date of birth, if born after this date. For each cancer site, the subjects in the cohort accrued person–years at risk until the date of diagnosis, if any, or until the date of death or until 31 December 1992, whichever occurred first. Seven parents whose dates of death were discordant in different sources, or who would otherwise be more than 100 years old at the end of follow-up, were contributing person–years up to the midpoint of the follow-up period. As a measure of association, we used the standardized incidence ratios (SIRs) and their exact 95% confidence interval (CI), assuming the observed number of events to follow a Poisson distribution (Breslow and Day, 1987). To calculate the expected cancer in the cohort, we used the national population age- and sex-specific incidence rates by quinquennia of age (0–4, 5–9 years etc.) and calendar period (1958–62, 1963–67, etc.). The rates in the period 1988–91 were also applied to the year 1992. For all cases of parathyroid tumours ($n$=4), the original notification to the Cancer Register was examined. In all instances, a diagnosis of adenoma was clearly stated. However, these cases are included in the calculation of the SIR for all cancers, because they contribute to the national incidence rates and, therefore, to the expected number of cancers as well.

 © Cancer Research Campaign 1997

**Table 2** Relative risk (OR) of thyroid cancer by parental history of any cancer in a matched case–control study, Uppsala, Sweden, 1980–93

| History of cancer (yes *vs* no) | Cases (*n*) | Controls (*n*) | OR | CI[b] |
|---|---|---|---|---|
| Any of the parents | 192 | 174 | 1.11 | 0.85–1.46 |
| Mother | 103 | 84 | 1.27 | 0.91–1.78 |
| Father | 109 | 105 | 1.05 | 0.77–1.43 |
| Both parents[a] | 20 | 15 | 1.39 | 0.69–2.79 |

[a]No = no cancer in any of the parents. [b]CI 95% confidence interval.

**Table 3** Relative risk of thyroid cancer by parental age at the proband's birth in a matched case–control study, Uppsala, Sweden, 1980–93

| | Cases (*n*) | Controls (*n*) | OR | CI[a] |
|---|---|---|---|---|
| Father's age (years) | | | | |
| ≤ 27 | 171 | 134 | 1 | Reference |
| 28–32 | 137 | 138 | 0.74 | 0.54–1.03 |
| 33–37 | 99 | 112 | 0.72 | 0.50–1.04 |
| 38+ | 91 | 114 | 0.60 | 0.41–0.88 |
| | | | $X_1^2$ trend=6.87 | (*P*=0.009) |
| Per year of increasing age | | | 0.98 | 0.96–1.00 |
| Mother's age (years) | | | | |
| ≤ 23 | 142 | 136 | 1 | Reference |
| 24–28 | 165 | 141 | 1.11 | 0.80–1.52 |
| 29–33 | 113 | 128 | 0.81 | 0.58–1.15 |
| 34+ | 89 | 108 | 0.78 | 0.53–1.14 |
| | | | $X_1^2$ trend=2.94 | (*P*=0.087) |
| Per year of increasing age | | | 0.98 | 0.96–1.00 |

[a]CI, 95% confidence interval.

### RESULTS

### Case–control study

The study encompassed 517 age- and gender-matched case–control sets. Table 1 gives information on the patients' age, gender and histological type of thyroid cancer.

#### *Relative risk of parental cancer*

No association was found between a parental diagnosis of cancer and risk of thyroid cancer (Table 2). Having a mother or both parents with a cancer seemed to convey a slightly increased risk, but could be owing to chance. There was an equal number of cases and controls (*n*=8) with a parent with a second diagnosis of primary cancer, after exclusion of diagnoses of parathyroid adenoma and of sites not specified as primary.

When the probands were analysed separately by sex and histopathological form, a positive association of borderline statistical significance was apparent between maternal cancer and thyroid cancer among women (OR 1.43, 95% CI 0.97–2.10), particularly those women with a diagnosis of follicular carcinoma (OR 2.11, 95% CI 0.96–4.67). This latter association was strengthened when the number of ascertained parents and the living status of the mother were included in the multivariate analysis (OR 2.35, 95% CI 1.03–5.37).

In the multivariate analysis, all the associations with parental history of cancer were not substantially altered after adjustment for parent's age at the proband's birth.

#### *Age of parents at the proband's birth*

Both parents of controls were, on average, older than parents of cases when their index child was born. When analysed in regression models, this difference resulted in a decreased risk with increasing quartiles of paternal and maternal age at the proband's birth, although the estimates attained a borderline statistical significance only for paternal age (Table 3). Paternal and maternal age, however, were strongly positively correlated (Pearson *r*=0.74). In a separate analysis using sex of the probands as a variable (not shown), these results held true and were even strengthened among women, but not among men. When parental age was analysed in the continuous form, women had a 3% significant reduction in risk for each year of increasing paternal age and maternal age (OR 0.97, 95% CI 0.95–0.99) at the time of their birth. The same tendency was seen in the subset of papillary carcinoma, while the analysis of follicular carcinoma was hampered by the paucity of the observations.

The results did not change after mutual adjustment by history of cancer in either parent or both.

### SIR of cancer among parents

There were 147 cases of cancer in the cohort of 778 parents of cases of thyroid cancer (SIR 1.05, 95% CI 0.88–1.23), contributing slightly more than 18 000 person–years at risk during the follow-up period.

Table 4 reports the relative risk of specific cancer sites (ICD7 3-digit classification) by sex of the parents. Increased risk, although not statistically significant, was found for malignant neoplasms of the thyroid gland, especially among women (SIR 3.54, 95% CI 0.73–10.36) who also had increased risk of stomach cancer (SIR 2.27, 95% CI 1.04–4.31) and of parathyroid adenomas.

There was also an excess risk for cancer of the nasopharynx among women and of the pharynx among men, the latter being statistically significant (SIR 69.36, 95% CI 1.76–386.47) but based on only one case in each instance. A significant excess of malignancies of nose and sinuses was also observed among males, with two observed cases *vs* 0.2 expected (Sir 9.78, 95% CI 1.18–35.32).

In this cohort the SIR was lower than expected for cancer of several sites. The most striking deficits occurred in lung cancer among men, with only one case observed *vs* 7.49 expected (SIR 0.13, 95% CI 0.00–0.74), in breast cancer among women (SIR 0.57, 95% CI 0.27–1.05) and in tumours of the pancreas in both sexes (SIR 0.18, 95% CI 0.00–1.03). Other deficits (although based on very few observed and/or expected cases) included: anorectal and ovary cancer among women and, in both sexes, malignant melanoma, non-Hodgkin lymphoma and plasmocytoma. These deficits were apparently not as the result of elevated early mortality for these malignancies, the proportions such deaths being practically identical between those parents of thyroid cancer cases and those parents of controls who died before 1958. A slight excess, yet largely compatible with chance, was found only for breast cancer among mothers of cases.

A separate analysis of incidence among parents of female probands (607 subjects) did not reveal any further difference, and likewise when the analysis was restricted to the 619 parents whose children were diagnosed with papillary carcinoma. However, in this latter group, the SIR for thyroid neoplasms was significantly higher than in the whole cohort (SIR 4.25, 95% CI 1.16–10.89).

© Cancer Research Campaign 1997

**Table 4** Standardized incidence ratio (SIR) of cancer at different sites in a cohort of parents of patients with papillary and follicular carcinoma of the thyroid, Uppsala, Sweden, 1958–92

| Site (Three-digit ICD7) | Both sexes Cases O | E | SIR | CI[a] | Males Cases O | E | SIR | CI[a] | Females Cases O | E | SIR | CI[a] |
|---|---|---|---|---|---|---|---|---|---|---|---|---|
| All sites (140–207)[b] | 147 | 140.3 | 1.05 | 0.88–1.23 | 77 | 70.82 | 1.09 | 0.86–1.36 | 70 | 69.53 | 1.01 | 0.78–1.27 |
| Lip (140) | 2 | 0.94 | 2.12 | 0.26–7.65 | 2 | 0.82 | 2.43 | 0.29–8.76 | 0 | 0.12 | 0.00 | 0.00–25.00 |
| Nasopharynx (146) | 1 | 0.21 | 4.71 | 0.12–26.22 | 0 | 0.13 | 0.00 | 0.00–23.07 | 1 | 0.08 | 12.36 | 0.31–68.84 |
| Pharynx, unspecified (148) | 1 | 0.02 | 46.61 | 1.18–259.69 | 1 | 0.01 | 69.36 | 1.76–386.47 | 0 | 0.01 | 0.00 | 0.01–300.0 |
| Oesophagus (150) | 1 | 1.46 | 0.69 | 0.02–3.82 | 1 | 1.01 | 0.99 | 0.03–5.51 | 0 | 0.45 | 0.00 | 0.00–6.67 |
| Stomach (151) | 16 | 10.67 | 1.50 | 0.86–2.43 | 7 | 6.71 | 1.04 | 0.42–2.15 | 9 | 3.97 | 2.27 | 1.04–4.31 |
| Colon (153) | 15 | 12.00 | 1.25 | 0.70–2.06 | 7 | 5.80 | 1.24 | 0.48–2.48 | 8 | 6.19 | 1.29 | 0.56–2.55 |
| Rectum and anus (154) | 6 | 6.96 | 0.86 | 0.32–1.88 | 6 | 3.96 | 1.51 | 0.56–3.30 | 0 | 3.00 | 0.00 | 0.00–1.00 |
| Biliary passages and liver, primary (155) | 4 | 4.77 | 0.84 | 0.23–2.15 | 0 | 2.08 | 0.00 | 0.00–1.44 | 4 | 2.69 | 1.49 | 0.41–3.81 |
| Liver, not specified (156) | 1 | 0.57 | 1.75 | 0.04–9.73 | 0 | 0.29 | 0.00 | 0.00–10.34 | 1 | 0.28 | 3.58 | 0.09–19.97 |
| Pancreas (157) | 1 | 5.41 | 0.18 | 0.00–1.03 | 1 | 2.86 | 0.35 | 0.01–1.95 | 0 | 2.55 | 0.00 | 0.00–1.18 |
| Nose and nasal sinuses (160) | 2 | 0.33 | 6.03 | 0.73–21.78 | 2 | 0.20 | 9.78 | 1.18–35.32 | 0 | 0.13 | 0.00 | 0.00–23.08 |
| Larynx (161) | 1 | 0.85 | 1.17 | 0.03–6.52 | 1 | 0.77 | 1.30 | 0.03–7.24 | 0 | 0.09 | 0.00 | 0.00–33.33 |
| Trachea, bronchus, lung and pleura (162) | 4 | 9.93 | 0.40 | 0.11–1.03 | 1 | 7.49 | 0.13 | 0.00–0.74 | 3 | 2.44 | 1.23 | 0.25–3.59 |
| Lung, not specified as primary (163) | 1 | 0.60 | 1.68 | 0.04–9.36 | 0 | 0.40 | 0.00 | 0.00–7.50 | 1 | 0.20 | 5.07 | 0.13–28.24 |
| Breast (170) | 11 | 17.70 | 0.62 | 0.31–1.11 | 1 | 0.13 | 7.67 | 0.19–42.71 | 10 | 17.57 | 0.57 | 0.27–1.05 |
| Cervix uteri (171) | – | | | | – | | | | 2 | 2.86 | 0.70 | 0.08–2.52 |
| Corpus uteri (172) | – | | | | – | | | | 6 | 4.04 | 1.49 | 0.55–3.24 |
| Ovary, tube and broad ligament (175) | – | | | | – | | | | 2 | 4.28 | 0.47 | 0.06–1.69 |
| Other female genitals (176) | – | | | | – | | | | 1 | 0.76 | 1.31 | 0.03–7.32 |
| Prostate (177) | – | | | | 22 | 17.52 | 1.26 | 0.79–1.90 | – | | | |
| Kidney (180) | 5 | 5.20 | 0.96 | 0.31–2.24 | 2 | 3.01 | 0.66 | 0.08–2.40 | 3 | 2.19 | 1.37 | 0.28–4.01 |
| Other urinary organs (181) | 5 | 6.66 | 0.75 | 0.24–1.75 | 5 | 4.85 | 1.03 | 0.34–2.41 | 0 | 1.81 | 0.00 | 0.00–1.66 |
| Malignant melanoma of the skin (190) | 1 | 2.81 | 0.36 | 0.01–1.99 | 0 | 1.31 | 0.00 | 0.00–2.29 | 1 | 1.49 | 0.67 | 0.02–3.73 |
| Skin, excluded melanoma (191) | 6 | 5.08 | 1.18 | 0.43–2.57 | 3 | 3.26 | 0.92 | 0.19–2.69 | 3 | 1.82 | 1.65 | 0.34–4.81 |
| Eye (192) | 1 | 0.39 | 2.57 | 0.06–14.29 | 1 | 0.20 | 4.97 | 0.13–27.70 | 0 | 0.19 | 0.00 | 0.00–15.79 |
| Nervous system (193) | 3 | 4.01 | 0.75 | 0.15–2.19 | 1 | 1.80 | 0.56 | 0.01–3.09 | 2 | 2.21 | 0.91 | 0.11–3.27 |
| Thyroid (194) | 4 | 1.19 | 3.35 | 0.91–8.59 | 1 | 0.35 | 2.89 | 0.07–16.11 | 3 | 0.85 | 3.54 | 0.73–10.36 |
| Other endocrine (195)[c] | 4 | 1.91 | 2.09 | 0.57–5.36 | 0 | 0.59 | 0.00 | 0.00–5.08 | 4 | 1.32 | 3.03 | 0.83–7.76 |
| Bone (196) | 1 | 0.22 | 4.50 | 0.11–25.07 | 1 | 0.12 | 8.54 | 0.22–47.59 | 0 | 0.11 | 0.00 | 0.00–27.27 |
| Connective tissue, muscle (197) | 1 | 0.93 | 1.08 | 0.03–6.00 | 1 | 0.48 | 2.09 | 0.05–11.64 | 0 | 0.45 | 0.00 | 0.00–6.67 |
| Non-Hodgkin lymphoma (200) | 1 | 3.36 | 0.30 | 0.01–1.66 | 1 | 1.78 | 0.56 | 0.01–3.13 | 0 | 1.58 | 0.00 | 0.00–1.90 |
| Hodgkin's disease (201) | 1 | 0.83 | 1.20 | 0.03–6.70 | 0 | 0.48 | 0.00 | 0.00–6.25 | 1 | 0.35 | 2.86 | 0.07–15.92 |
| Plasmacytoma (203) | 1 | 2.15 | 0.46 | 0.01–2.59 | 0 | 1.17 | 0.00 | 0.00–2.56 | 1 | 0.98 | 1.02 | 0.03–5.66 |
| Lymphatic leukaemia (204) | 4 | 2.85 | 1.40 | 0.38–3.59 | 4 | 1.69 | 2.36 | 0.64–6.05 | 0 | 1.16 | 0.00 | 0.00–2.59 |
| Myeloid leukaemia (205) | 1 | 0.81 | 1.24 | 0.03–6.90 | 1 | 0.41 | 2.42 | 0.06–13.49 | 0 | 0.39 | 0.00 | 0.00–7.69 |
| Other/unspecified site (199) | 8 | 4.73 | 1.69 | 0.73–3.34 | 4 | 2.14 | 1.87 | 0.51–4.78 | 4 | 2.58 | 1.55 | 0.42–3.97 |

[a]CI, exact 95% confidence intervals. [b]Only sites with at least one observed or expected case are included in the table. For each specific site, person–time at risk was contributed also from subjects with other cancer diagnoses. Therefore, the sum of expected cases at specific sites does not equal the number of expected cancer at all sites. [c]Parathyroid adenomas. O, observed; E, expected.

**Table 5** Standardized incidence ratios (SIRs) of selected parental cancer sites by histopathological type of the probands

| Site/Parents | Papillary carcinoma O | E | SIR | 95% CI | Follicular carcinoma O | E | SIR | 95% CI |
|---|---|---|---|---|---|---|---|---|
| All sites/All | 107 | 108.4 | 0.99 | 0.81–1.19 | 40 | 31.93 | 1.25 | 0.90–1.71 |
| Corpus uteri/Mothers | 3 | 3.23 | 0.93 | 0.19–2.71 | 3 | 0.81 | 3.72 | 0.77–10.87 |
| Breast/Mothers | 7 | 13.91 | 0.50 | 0.20–1.04 | 3 | 3.66 | 0.82 | 0.17–2.40 |
| Pancreas/All | 0 | 4.08 | 0.00 | 0.00–0.74 | 1 | 1.33 | 0.75 | 0.02–4.20 |
| Colon/All | 13 | 9.02 | 1.44 | 0.77–2.47 | 2 | 2.98 | 0.67 | 0.08–2.43 |
| Stomach/Mothers | 5 | 2.87 | 1.74 | 0.57–4.06 | 4 | 1.10 | 3.65 | 0.99–9.35 |
| Thyroid/All | 4 | 0.94 | 4.25 | 1.16–10.89 | 0 | 0.25 | 0.00 | 0.00–12.00 |
| Thyroid/Mothers | 3 | 0.67 | 4.49 | 0.93–13.12 | 0 | 0.18 | 0.00 | 0.00–16.67 |

O, observed; E, expected.

© Cancer Research Campaign 1997

All of the four observed cases of thyroid cancer occurred among parents of probands with papillary carcinoma. In three of these four parent–child couples, we have secure information on the histotype of thyroid cancer for the parent, as both parent and child were included as probands in our case series. In two instances, the parent also had papillary carcinoma, while in the third couple the parent had follicular carcinoma. The excess risk of stomach cancer, on the other hand, was evidence only among mothers whose children were diagnosed with a follicular carcinoma (SIR 3.65, 95% CI 0.99–9.35). In Table 5, some selected results are presented separately by histopathological type of the proband.

### DISCUSSION

An overall history of cancer in the parents was not linked to the risk of differentiated non-medullary carcinoma of the thyroid in this large, population-based case–control study. However, some associations emerged, though of marginal significance, between certain histotypes of thyroid cancer and parental cancer at specific sites. The risk of follicular carcinoma among women was associated with a history of maternal cancer. Compared with the general population, mothers of patients with follicular thyroid carcinoma had an increased risk of stomach cancer, while the risk of having thyroid cancer was higher among parents whose children had papillary carcinoma.

This last finding is in agreement with previous observations (Ozaki et al, 1988; Fischer et al, 1989; Ron et al, 1991; Gorson et al, 1992; Kobahashi et al, 1995). As in some studies (Ron et al, 1991; Kobayashi et al, 1995), we did not observe a complete concordance between the tumour histotype of the child and that of the parent. The role of increased medical surveillance in these families cannot be ruled out; on the other hand, the association was quite strong, and our estimate of a fourfold increase in risk might even be conservative if, in these families, differentiated non-medullary thyroid carcinoma occurred at a younger age than in the general population, but had the same overall good prognosis (Akslen et al, 1991; Levi et al, 1992). The excess of parathyroid adenomas among mothers of thyroid cancer cases deserves attention. Again, chance and increased diagnostic intensity are plausible explanations, but a multiple endocrine involvement in these families is also possible.

The increased risk of stomach cancer among mothers is intriguing, if real. An expression, at the maternal lineage, of familial polyposis coli (FAP) syndromes (Plail et al, 1987) does not seem likely, as the incidence of colorectal tumours was close to the expected, and FAP was more often associated with papillary carcinoma. None of the gastric tumours occurred in association with other tumours, and the incidence of pancreatic cancer, which is increased in patients with FAP (Giardiello et al, 1993), was even decreased in this cohort of parents compared with the general population. Although not well recognized, a linkage has been proposed between iodine deficiency and carcinogenesis of the stomach (Venturi et al, 1993). Thyroid cancer has been repeatedly associated with iodine deficiency disorders, such as goitres and thyroid nodules (Ron et al, 1987; Preston-Martin et al, 1987; D' Avanzo et al, 1995) and with residence in areas of endemic goitre (Franceschi et al, 1989). Shared dietary habits among members of these families could, at least in part, explain this finding. A gene–environment interaction (Schatzkin et al, 1995) can also be hypothesized, if a putative genetic defect might induce an altered cell response to iodine deficiency or alter iodine metabolism in several target organs (e.g. thyroid and stomach). The plausibility of a link with iodine deficiency may be increased given that the excess of stomach cancer appeared among mothers of probands with follicular carcinoma. The incidence of this histological type seems to be relatively high in areas of iodine deficiency (Belfiore et al, 1987; Franssila et al, 1981), and women may be more sensitive to an insufficient iodine intake (Galanti et al, 1995).

The risk deficit of cancers at specific sites may be owing to chance, given the high number of tested associations. Alternatively, it may reflect the selection of 'healthy reproducers' and 'healthy survivors', but one would then expect a deficit of incidence for all cancers. The use of national data in the calculation of the expected number of cancers might also explain to a very small extent the deficits of lung and breast cancer, if the regional rates are lower than the national ones. The deficit of lung cancer among fathers may indicate that smoking is uncommon in these families. In previous case–control studies, there was a lower proportion of women smokers among thyroid cancer cases (Hallquist et al, 1993) or among their mothers (Paoff et al, 1995) compared with their corresponding controls. Familial environment may influence the uptake of smoking habits (Evans et al, 1995). Tobacco smoking is associated to pancreatic cancer (Silverman et al, 1994), another cancer site of which there was a decreased incidence.

A familial association of breast and thyroid cancer was detected in a study of first-degree relatives (Goldgar et al, 1994), but not in other epidemiological studies (Ron et al, 1987, McTiernan et al, 1987). Our cohort consisted, by definition, of parous women only, and this may account for the decreased risk of breast cancer (Lambe et al, 1996), paralleled by a concomitant deficit of ovarian cancer (Adami et al, 1994). The possibility that an excess of incidence of breast cancer occurred, in this cohort, at a younger age, cannot be ruled out; however, if the protection conferred by parity was counteracted by an increased familial risk, the net effect would not have been the marked reduction in risk that we observed.

The decreased risk of differentiated thyroid carcinoma among women with increasing parental age at their birth is not easily explainable. If not an artifact or owing to chance, high parental age may reflect social class or birth order of the index child, with possible differences in nutritional factors or exposure to lower maternal pregnancy hormones (Panagiotopoulou et al, 1990). Although this was not the focus of our study, this finding deserves further attention, in the light of the linkage between hormonal and metabolic factors and thyroid cancer among women (Goodman et al, 1992).

This study has an inherent limitation in the information about the diagnosis of cancer among the oldest parents, because death records may lack both sensitivity and specificity. A more complete family history of cancer including all first-degree relatives could not be compiled at this stage. The strength of this study, on the other hand, rests on several features: the thorough and uniform ascertainment and histological verification of a large series of thyroid cancer cases, the population-based design and the retrieval of information on parental cancer free from selection and recall bias.

Our findings provide insights and indications for future research in two directions. Firstly, they support the existing evidence that some familial, possibly genetically inheritable, factors are associated with differentiated, non-medullary thyroid carcinoma. Secondly, the role of common environmental factors and lifestyles is suggested in the occurrence of familial associations of thyroid cancer with other malignant neoplasms.

### ACKNOWLEDGEMENTS

We thank Ann Almqvist and Monica Rundgren for assistance in the data collection; Matthew Zack, PhD, MD, Center for Diseases

© Cancer Research Campaign 1997 

Control, Atlanta, GA, USA for methodological suggestions and Pär Sparén, PhD, Department of Cancer Epidemiology, Uppsala, for assistance in the data analysis. We are also indebted to the administrative officers of the Swedish parishes for their collaboration. This work was partly supported by grant no. 3136-B92-02XBB from the Swedish Cancer Society and by grant no. 102 from the Swedish Medical Research Council.

### REFERENCES

Adami HO, Hsieh C-C, Lambe M, Trichopoulos D, Leon D, Persson I, Ekbom A and Janson PO (1994) Parity, age at first birth and risk of ovarian cancer. *Lancet* **344**: 1250–1254

Akslen LA, Haldorsen T, Thoresen S and Glattre E (1991) Survival and causes of death in thyroid cancer: a population-based study of 2479 cases from Norway. *Cancer Res* **51**: 1234–1241

Belfiore A, LA Rosa G, Padova G, Sava L, Ippolito O and Vigneri R (1987) The frequency of cold thyroid nodules and thyroid malignancies in patients from an iodine-deficient area. *Cancer* **60**: 3096–3102

Breslow NE and Day NE (1980) *Statistical Methods in Cancer Research*. Vol.1 *The Analysis of Case–Control Studies* IARC: Lyon

Breslow NE and Day NE (1987) *Statistical Methods in Cancer Research*. Vol.II. Heseltine E (ed.), *The Design and Analysis of Cohort Studies* IARC: Lyon

D'avanzo B, La Vecchia C, Franceschi S, Negri E and Talamini R (1995) History of thyroid diseases and subsequent thyroid cancer risk. *Cancer Epidemiol Biomarkers Prev* **4**: 193–199

Evans N, Farkas A, Gilpin E, Berry C and Pierce JP (1995) Influence of tobacco marketing and exposure to smokers on adolescent susceptibility to smoke. *J Natl Cancer Inst* **87**: 1538–1545

Fischer DK, Groves MD, Thomas SJ and Johnson PC (1989) Papillary carcinoma of the thyroid: additional evidence in support of a familial component. *Cancer Invest* **7**: 323–325

Franceschi S, Fassina A, Talamini R, Mazzolini A, Vianello S, Bidoli E, Serraino D and La Vecchia C (1989) Risk factors for thyroid cancer in Northern Italy. *Int J Epidemiol* **18**: 578–584

Franssila K, Saxén E, Teppo L, Bjarnason O, Tulinius H, Normann T and Ringertz N (1981) Incidence of different morphological types of thyroid cancer in the Nordic Countries. *Acta Pathol Microbiol Scand* A, **89**: 49–55

Galanti MR, Sparén P, Karlsson A, Grimelius L and Ekbom A (1995) Is residence in areas of endemic goiter a risk factor for thyroid cancer? *Int J Cancer* **61**: 615–621

Giardiello FM, Offerhaus GJA, Lee DH, Krush AG, Tersmette AC, Booker SV, Kelley NC and Hamilton SR (1995) Increased risk of thyroid and pancreatic carcinoma in familial adenomatous polyposis. *Gut* **34**: 1394–1396

Goldgar D, Easton DF, Cannon-Albright L and Skolnick MH (1994) Systematic population-based assessment of cancer risk in first-degree relatives of cancer probands. *J Natl Cancer Inst* **86**: 1600–1607

Goodman MT, Kolonel LN and Wilkens LR (1992) The association of body size, reproductive factors and thyroid cancer. *Br J Cancer* **66**: 1180–1184

Gorson D (1992) Familial papillary carcinoma of the thyroid. *Thyroid* **2**: 131–132

Hallquist A, Hardell L, Degerman A and Boquist L (1993) Occupational exposures and thyroid cancer: results of a case–control study. *Eur J Cancer Prev* **2**: 345–349

Hedinger C, Williams ED and Sobin LH (1988) Histological typing of thyroid tumours, In *International Histological Classification of Tumors*. 2nd edn. World Health Organization. Springer-Verlag: Berlin

Kobayashi K, Tanaka Y, Ishiguro S, Mori T, Mitani Y and Shigemasa C (1995) Family with nonmedullary thyroid neoplasms. *J Surg Oncol* **58**: 274–277

Lambe M, Hsieh C-C, Chan H-W, Ekbom A, Trichopoulos D and Adami HO (1996) Parity, age at first and last birth and risk of breast cancer. A population-based study in Sweden. *Breast Cancer Res Treat* **38**: 305–311

Levi F, Randimbison L, Te V-C, Franceschi S, and La Vecchia C (1992) Trends in cancer survival in Vaud, Switzerland. *Eur J Cancer* **28A**: 1490–1495

Li Volsi VA and Asa SL (1994) The demise of follicular carcinoma of the thyroid gland. *Thyroid* **4**: 233–236

Love RR, Evans AM and Josten DM (1985) The accuracy of patient reports of a family history of cancer. *J Chronic Dis* **38**: 289–293

McTierman A, Weiss NS and Daling JR (1987) Incidence of thyroid cancer in women in relation to known or suspected risk factors for breast cancer. *Cancer Res* **47**: 292–295

Ozaki O, Ito K, Kobayashi K, Suzuki A, Manabe Y and Hosoda Y (1988) Familial occurrence of differentiated, non medullary, thyroid carcinoma. *World J Surg* **12**: 565–571

Panagiotopoulou K, Katsouyanni K, Petridou E, Garas Y, Tzonou A and Trichopoulos D (1990) Maternal age, parity and pregnancy estrogens. *Cancer Causes Control* **1**: 119–124

Paoff K, Preston-Martin S, Mack WJ and Monroe K (1995) A case-control study of maternal risk factors for thyroid cancer in young women (California, United States). *Cancer Causes Control* **6**: 389–397

Plail RO, Bussey HJR, Glazer G and Thomson JPS (1987) Adenomatous polyposis: an association with carcinoma of the thyroid. *Br J Surg* **74**: 377–380

Preston-Martin S, Bernstein L, Pike MC, Maldonado AA and Henderson BE (1987) Thyroid cancer among young women related to prior thyroid disease and pregnancy history. *Br J Cancer* **55**: 191–195

Ron E, Kleinermann RA, Boice JD JR, Li Volsi VA, Flannery JT and Fraumeni JF JR (1987) A population-based case–control study of thyroid cancer. *J Natl Cancer Inst* **79**: 1–12

Ron E, Kleinerman RA, Livolsi VA and Fraumeni JF JR (1991) Familial nonmedullary thyroid cancer. *Oncology* **48**: 309–311

Schatzkin A, Goldstein A and Freedman LS (1995) What does it mean to be a cancer gene carrier? Problems in establishing causality from the molecular genetics of cancer. *J Natl Cancer Inst* **87**: 1126–1130

Silverman DT, Dunn JA, Hoover RN, Schiffman M, Lillemoe KD, Schoenberg JB, Brown LM, Greenberg RS, Hayes RB, Swanson GM, Wacholder S, Schwartz AG, Liff JM and Pottern LM (1994) Cigarette smoking and pancreas cancer: a case control study based on direct interviews. *J Natl Cancer Inst* **86**: 1510–1516

Tepper A, Connally LB, Haltmeier P, Smith E and Sweeney MH (1993) Knowledge of medical history information among proxy respondents for deceased study subjects. *J Clin Epidemiol* **46**: 1243–1248

Venturi S, Venturi A, Cimini D, Arduini C, Venturi M and Guidi A (1993) A new hypothesis: iodine and gastric cancer. *Eur J Cancer Prev* **2**: 17–23

© Cancer Research Campaign 1997